# Supplementary figures and images for: A study on the characteristics of coke in the hearth of a superlarge blast furnace
Source: PLoS One. 2021 Mar 3;16(3):e0247051. doi: 10.1371/journal.pone.0247051 (PMC7928459; doi:10.1371/journal.pone.0247051)

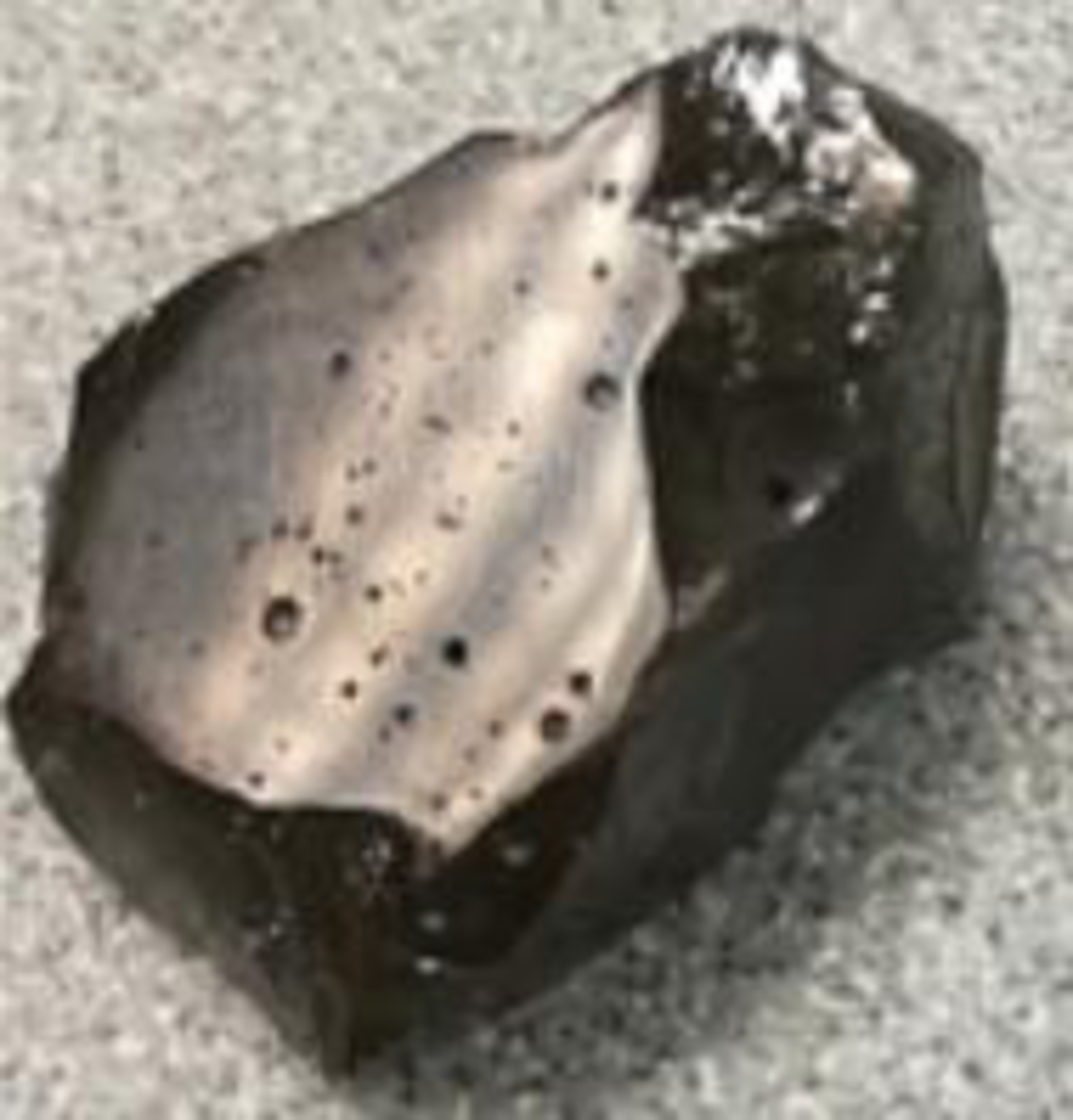

Supplement: S1 Fig — (TIF) [file pone.0247051.s001.tif]

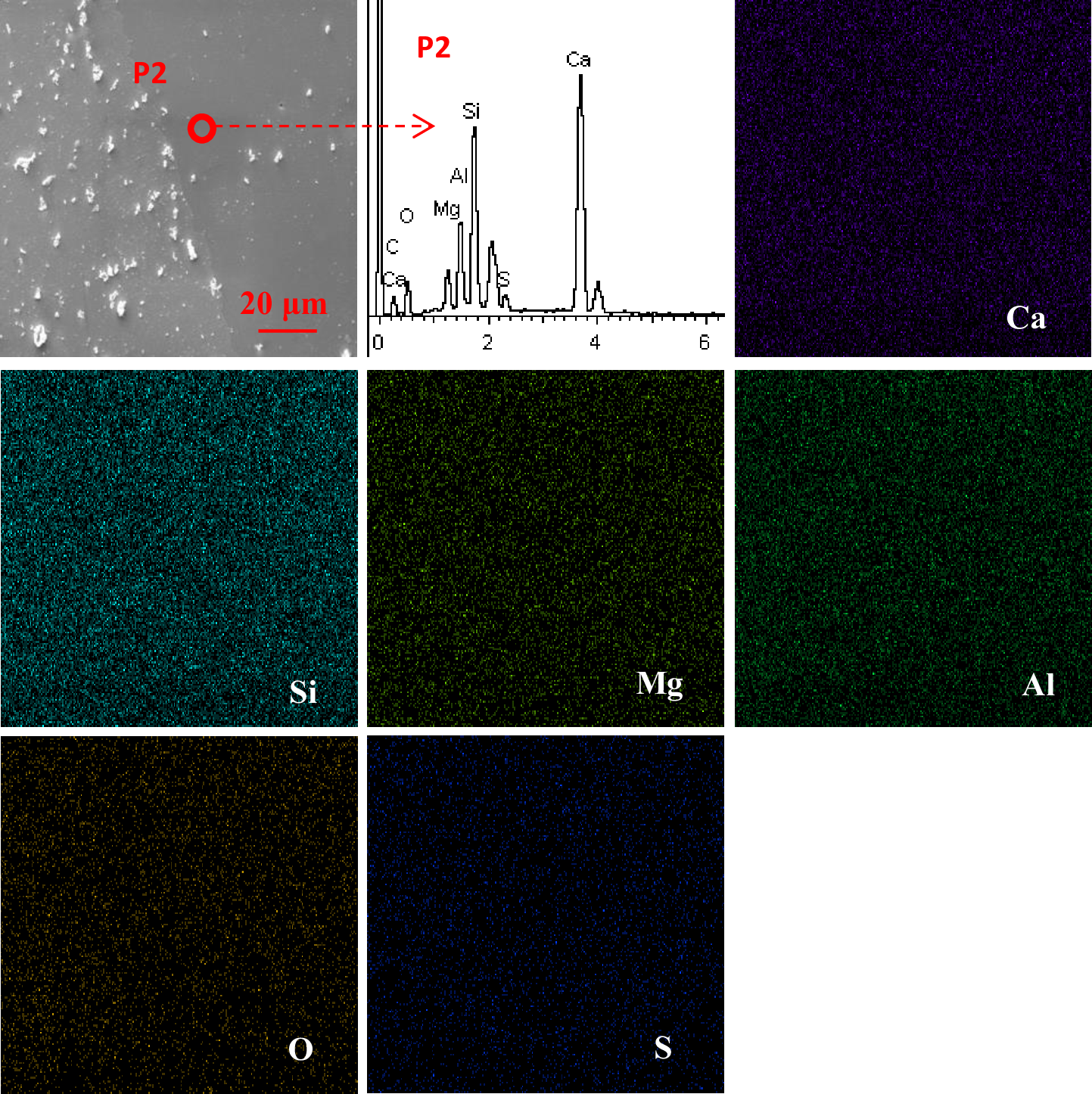

Supplement: S2 Fig — The results of an energy spectrum analysis show that the main components of blast furnace slag are Ca, Al, Mg, Si, and O, which is consistent with the results in the references. A small amount of S was also found in the slag. S comes from coke, and sulfur is absorbed by blast furnace slag. (TIF) [file pone.0247051.s002.tif]
