# Supplementary material for: A study on the characteristics of coke in the hearth of a superlarge blast furnace
Source: PLoS One. 2021 Mar 3;16(3):e0247051. doi: 10.1371/journal.pone.0247051 (PMC7928459; doi:10.1371/journal.pone.0247051)
Supplement: S1 File — (DOCX) [file pone.0247051.s003.docx]

**Cover letter**

Journal: PLOS ONE

Title: PhD. Candidate

Dear editor/reviewers,

We submit our manuscript entitled “***A Study on the Characteristics of Coke in the Hearth of a Superlarge Blast Furnace***” to PLOS ONE.

Coke serves as the structural support, fuel, reducing agent, and carburizer in blast furnaces. As the iron-making technologies advance, blast furnaces tend to be larger with higher rates of pulverized coal injection (PCI), a trend that raises higher requirements for the coke quality. As a response, exploring the mechanism of coke deterioration in blast furnaces is important for developing iron-making technologies. In the blast furnace process, high temperature and high pressure with extremely hostile conditions make it impossible to monitor the reactions that coke is involved in the furnace. This has hugely limited researchers’ understanding of the deterioration mechanism of coke in blast furnaces. With the tuyere sampling technique, researchers have obtained samples from operating blast furnaces and performed some basic studies on the structure, mineral transformation, reactivity, and post-reaction strength of coke, and improved the understanding of the process that coke deteriorates in the lower bottom part of a furnace. However, the sample amount, sampling location, and sampling depth are all restricted because it requires a sampling tube to be inserted into the blast furnace; and it is impossible to get all representative samples from the feedstock in the lower bottom part of the hearth. As for the 5,800 m3 super large blast furnace concerned in the present work, it is impossible to reach the central part of the deadman by using tuyere sampling. In addition, tuyere sampling is also limited to the horizontal direction at the tuyere level and unable to reach the coke below it, which prevents researchers from learning about the deterioration mechanism of the coke below tuyere level. In this paper, with the coke obtained from a 5,800 m3 super large blast furnace of Shagang Group, China during its intermediate repair, instruments including a polarizing microscope, X-ray diffraction (XRD) analyzer and a scan electronic microscope (SEM) were used to systematically characterize macro-morphology, and microstructure of the coke, in an effort to explore the deterioration mechanism of coke in the hearth of the blast furnace.

This study on the characteristics of coke in the hearth of blast furnaces is of great significance for explaining the mechanism of deterioration of coke in blast furnaces.

Neither the entire paper nor any part of its content has been published or has been accepted elsewhere. It is not being submitted to any other journal. We hope the manuscript could be considered for publication in PLOS ONE. Thank you very much for your reading. We are looking forward to hearing from you.

- **Language editing**

This manuscript is edited by American Journal Experts (AJE).

- **Funding Statement**

This work was only financially Supported by National Key R&D Program of China (2017YFB0304300&2017YFB0304303). The funder is National Key R&D Program of China. And no sponsors of funder play any role in the study design, data dollection and analysis, desision to publish, or preparation of the manuscript. The National Key R&D program provided support in the form of salaries for authors [QQL, YST, PD, JLZ and GHW], but did not have any additional role in the study design, data collection and analysis, decision to publish, or preparation of the manuscript. The specific roles of these authors are articulated in the ‘author contributions’ section.”

- **Competing Interests Statement**

The authors have read the journal's policy and the authors of this manuscript have the following competing interests: Authors YST and GHW are paid employees of The Key Laboratory of Hubei Province for Coal Conversion and New Carbon Materials. PD and JLZ are paid employee of Iron and Steel Research Institute of Shasteel. QQL is an paid employee of Jiangsu Huade Hydrogen Energy Technology Co., Ltd. At the same time, QQL is a doctoral student studying in Wuhan University of science and technology. There are no patents, products in development or marketed products to declare. This does not alter our adherence to PLOS ONE policies on sharing data and materials.

The Key Laboratory of Hubei Province for Coal Conversion and New Carbon Materials formerly known as the school-level key laboratory of deep processing of coal, coke and chemical products of Wuhan University of Science and Technology, was approved by the Hubei Provincial Department of Education in 2006 to formally establish a project to build Hubei Province coal conversion and new carbon materials. Focus on experimentation.

Iron and Steel Research Institute of Shasteel, Zhangjiagang is a provincial key research and development institution approved by the Jiangsu Provincial Government and built on Jiangsu Shagang Group.

Jiangsu Huade Hydrogen Energy Technology Co., Ltd. is a company engaged in the research and development of new energy equipments.

Best regards,

Sincerely

Qing-qing Lv

School of Chemistry and Chemical Engineering, Wuhan University of Science and Technology, Wuhan, Hubei, China
