# Supplementary material for: A study on the characteristics of coke in the hearth of a superlarge blast furnace
Source: PLoS One. 2021 Mar 3;16(3):e0247051. doi: 10.1371/journal.pone.0247051 (PMC7928459; doi:10.1371/journal.pone.0247051)
Supplement: Attachment — Submitted filename: Response to reviewers.docx [file pone.0247051.s004.docx]

Response to Reviewers

Dear two reviewers,

It is a great honor to receive the comments on this manuscript. Thank you for your patience. All the comments are very professional. I learned a lot from it. I have revised the manuscript to meet the requirements and the format of PLOS ONE. And I have chosen AJE for language editing of my manuscript. I hope that the resubmitted manuscript can meet the requirements of PLOS ONE.

If the manuscript still needs to be improved, please give feedback in time, and I will make corresponding modifications.

Thank you very much. The following is my reply to the two reviewers.

Reviewer #1:

The manuscript reports valuable data on the characteristics of the hearth coke samples from a very large blast furnace. This type of data are rare to obtain in the literature.
A major drawback of the work is that the manuscript focuses too much on the coke samples themselves without linking their characteristics to the reactions in a blast furnace, that is the effects of gasification and combustion in the raceway.

Answer:

Many thanks!

The gasification and combustion of coke in blast furnace are important reactions. The tuyere zone is the most important place for coke combustion and gasification. The gasification and combustion reactions of tuyere coke have been studied in a large number of papers, and the reaction mechanism is clear and definite. On the one hand, predecessors have carried out a lot of research on this issue, and the research on tuyere coke in this manuscript is not repeated. On the other hand, the study of coke in the tuyere zone of the manuscript will take up a lot of space, resulting in the length of this manuscript is too long. Therefore, the coke in tuyere area has not been studied and explained. This manuscript focuses on the study of coke properties at locations where are not accessible by tuyere sampling technology. Due to the huge volume of 5800m3 blast furnace and the limitation of blast furnace operation conditions, the deterioration mechanism of coke at the edge and center of blast furnace is different from that of small blast furnace.

(1) Figs. 15 and 17 are not found in the submission.

Answer:

Many thanks!

Sorry, the Figs. 15 and 17 missing in the upload process. Upload the image again, as shown in the figure below.

(2) Figure 1, the current marking of distances from the tuyere level should be positive values, not negative. The marking of distances 5.2 m and 4.4 m external of the furnace hearth section is unnecessary.

Answer:

Many thanks!

I've modified the picture.

(3)Figure 4, schematic demonstration

Answer:

Many thanks!

An explanation is added to the diagram.

(4)Figure 6 shows the change in mean particle size with location. How is the mean particle size calculated? The authors state that “The extremely high content of the coke powder in the hearth center significantly decreases the arithmetic mean particle size of the coke in this area.” In the view of the reviewer, the fine particles with size <10 mm should be excluded from the calculation to improve the certainty (that is, what value is used for the fine particles with particle size <10 mm?).

Answer:

Many thanks!

The average particle size of coke is affected by sampling method and sampling tools. The sampling site at that time is shown in the figure below. Both reviewers raised questions about the average size of coke.

As a part of the samples have been used, the remaining samples can not be retested for the average particle size of coke. Therefore, in order to avoid misleading readers with wrong conclusions, this part of the data is deleted.

（5）The authors state that “As shown in Fig.9(b), the intensity of (002) peak increases as the sampling location moves toward the hearth center, an indicator of the increased degree of graphitization.” “Increases” and “increased” should be decreased here to match the results.
Answer:

Many thanks!

This sentence is wrong. Make corresponding modification according to the conditions.

“As shown in Fig.9(b), the intensity of (002) peak increases as the sampling location moves toward the hearth center, an indicator of the decreased degree of graphitization.”

(6)The authors discussed the difference in the degree of graphitisation of coke samples from 0 m and 2 m. This discuss may not be sensible because of a strong mixing of coke particles within raceway. The difference is minor and can be within experimental error.

Answer:

Many thanks!

It is possible that he difference is experimental error. According to your comments, make the following changes to the article. The original explanation of the manuscript is deleted and this part is discussed according comments as follwoing.

“It can also be seen from Fig.9 (b) that the graphitization degree of the coke at 0 m and 2 m away from the hearth edge is higher than that at a horizontal distance of 4m, 6m and 7.5m. The hearth edge is close to the tuyere zone where a large amount of heat is produced by coke combustion; as a result, the degree of graphitization of the coke along the edge is higher than that of the coke in the center. The difference in the degree of graphitisation of coke samples from 0 m and 2 m is minor and can be within experimental error, because of a strong mixing of coke particles within raceway.”

(7)Figures 9 and 10: The labelling of mineral phase Ca2MgSi2O7 is incorrect.

Answer:

Many thanks!

I made a correction to the label of the picture.

(8)In relation to the discussion in section 3.5, separate XRD analyses of the coarse and fine coke particles and differentiation of their degree of graphitisation help understanding of the source of fine coke particles, especially for samples 5 to 8.

Answer:

Many thanks!

I tried to analyze the source of fine powder in the deadman of blast furnace. The composition of fine powder in the deadman is very complex. There are mineral powder, coke powder, slag and iron powder. It is difficult to separate several components. An attempt has been made to separate the iron content in it with a magnet. However, the powder of the dead column was completely absorbed. Iron powder and coke powder have been fully mixed. It's very difficult to separate. Therefore, it is difficult to distinguish the source of the powder in the dead column. The picture is a picture of me trying to separate the powder from the dead column. Since the discussion of coke size has been deleted in this paper. Therefore, this part will not discuss coke powder.

(9)With regards to the discussion on Fig. 11 by SEM analysis, it is not clear how the images are representative of a whole coke sample,

Answer:

Because of the magnification of SEM is limited, the whole coke sample can not be scanned. In this manuscript, the maximum cross section of coke is scanned by scanning electron microscope and optical microscope as the internal structure of coke is shown in fig. 11 (h), fig. (13) and fig. 7 (j). In addition, this manuscript attempts to analyze the slag inside the coke for many times. Moreover, the optical properties of slag in different parts have been shown in fig. 7 (j), and the results show that the composition and properties of slag are consistent.

(10)and whether the authors tried to distinguish between the coke ash and the intruded bulk slag within coke particles.

Answer:

Many thanks!

In order to distinguish the slag between the coke as and the intruded bulk slag within coke particles, I tested the sample of fig.13 again. I tried to enlage the magnification and analysis the slag composition by EDS. It is found that there is a large amount of Si aggregation in the edge of the inner slag. This may be due to ash content in the coke. At the same time, it is found that there is Fe on the surface of the slag, which may be the form of FeO contained in the slag. Therefore, the manuscript was re analyzed and revised.

The manuscript is amended as follows:

The area-scan images of the coke at 7.5 m horizontal and -5.2 m vertical under EDS are shown in Fig.13. Major elements of slag are Ca, Si, Al, Mg, and O, a result that is consistent with the XRD patterns. Zhiyu Chang [23] found that slag is detected in coke pores in the cohesive zone of the blast furnace. The major elements of slag are also Ca, Si, Al, Mg, O. It can be inferred that the process of the slag in the coke enterring into the coke begins from the cohesive zone of the blast furnace. With the renewal of the deadman, the slag inside the coke goes down to the hearth of the blast furnace hearth together with the coke. Interestingly, the Si content at the edge of the slag in the pores is higher than that in the interior. This may be due to the formation of coke ash after coke gasification. The Si content in coke is significantly higher than that in blast furnace slag. The ash content in the surface layer of coke pores is considered to hinder coke gasification and slag erosion[25]. At the same time, a small amount of Fe was found at the edge of the slag, which may be a small amount of FeO in the slag.

**Fig.13 EDS images of the coke at 7.5 m horizontal and -5.2 m vertical**

(11) On Fig. 12, “major elements of the coke are O, Al, Ca, Fe, and Si”?

Answer:

Many thanks! This sentence is wrong. I revise the manuscript as follows:

“The EDS analysis of the coke at 2 m away from the hearth edge is shown in Fig.12. Major elements of the coke is C. And O, Al, Ca, Mgand Si are the major elements of the ash contained in the coke.”

(12)Current observation presented in Fig. 12 does not convincingly connect the presented Fe with metal phase. It is necessary to magnify the zone and determine the composition of the related phase by EDS to confirm that a metallic phase is present, rather than FeO in the slag.

Answer:

Many thanks!

As the sample in fig. 12 has been damaged, the energy spectrum analysis can not be continued. I took the coke sample in the same position as the original sample of fig.12 for SEM analysis. This sample can clearly see the morphology of liquid metal invading the surface of coke.

Figure 1 shows the appearance of the sample before testing. It can be seen from the surface layer of coke that there are traces of metal erosion on the surface of coke. Fig. 2 shows the SEM image of the sample. It can be seen from Figure 12 that the liquid metal erodes the surface of coke, and the surface of the eroded coke is smooth. The energy spectrum analysis of coke shows that the edge of coke is Fe, which is oxidized state. This is because Fe is exposed to the air for a long time and is oxidized to the oxidized state of iron. The test results are consistent with Fig. 1. There are a lot of Fe aggregation forms in the pores (P1) at the edge of coke. The energy spectrum analysis of P1 shows that Fe is the main one. It shows that a small amount of liquid metal can enter the pores of coke and penetrate into the surface of coke.

Based on the above analysis, the manuscript is revised as follows:

“The EDS analysis of the coke at 2 m away from the hearth edge is shown in Fig.12. Major elements of the coke is C. And O, Al, Ca, Mgand Si are the major elements of the ash contained in the coke. The edge of the liquid metal is eroded. Iron element, such as Fig. 12 (P1), was found in the surface pores of coke. As indicated in Fig.3(i), surfaces of the coke at 2 m away from the hearth edge directly contacts and gets eroded by the liquid metal. It is generally believed that coke plays a carburizing role for the liquid metal. it is also found from Fig.12 that molecules in the liquid metal can penetrate into the coke through surfaces on which they contact the coke. ”

Fig. 1 apparent morphology

**Fig.12 EDS images of the coke at 2 m horizontal and -1 m vertical**

Reviewer #2: This manuscript investigated the mechanism of coke degradation in the blast furnace caused by its interaction with liquid iron and slag. This topic is interesting and importance for the steelmaking industry. However, major revision is required before publishing this manuscript as a research paper in PLOS ONE. The detailed comments are shown as below:

1. The introduction of this manuscript needs to be significantly improved. It needs to cover an introduction of research background, current development of this area, what have been done in the previous studies, what have not been fully investigated previously, and the importance of the investigation in this manuscript. The current one paragraph introduction is far from enough.

Answer:

Many thanks!

The manuscript is revised as requirtments:

Coke serves as the structural support, fuel, reducing agent, and carburizer in blast furnaces [1,2]. As the iron-making technologies advance, blast furnaces tend to be larger with higher rates of pulverized coal injection (PCI), a trend that raises higher requirements for the coke quality [3]. As a response, exploring the mechanism of coke deterioration in blast furnaces is important for developing iron-making technologies. In the blast furnace process, high temperature and high pressure with extremely hostile conditions make it impossible to monitor the reactions that coke is involved in the furnace. This has hugely limited researchers’ understanding of the deterioration mechanism of coke in blast furnaces. Huiqing Tang[4], Zhen Miao[5] and Yuting Zhou[6] established a mathematical simulation model to study the combustion behavior of coal and coke in the blast furnace tuyere zone. Gerd Rantitsch[7] found that Thermo-chemical reactivity and strength of blast furnace coke at 1100 ℃ is depend on the state of graphitized of the feed coke. Samples taken from the tuyere area of the blast furnace show that carbonaceous matter graphitization occurs at temperatures above 1900 ° C. Zhang’s[8] research shows that the physical properties of aluminosilicate in coke have hardly changed in the process of coke gasification in blast furnace, but they combine with alkali. The iron bearing phase has high activity and strong interaction with carbon matrix. Iron was proven to have a strong catalytic effect of iron on graphitization in the temperature range of 1100 °C–1500 °C. [3-11] With the tuyere sampling technique, researchers have obtained samples from operating blast furnaces and performed some basic studies on the structure [12-15], mineral transformation [16-20], reactivity, and post-reaction strength of coke [21,22], and improved the understanding of the process that coke deteriorates in the lower bottom part of a furnace. Zhiyu Chang’s research shows that the pores in the coke of the cohesive zone of the blast furnace are filled with slag. The main composition of slag is chlorite or anorthite. Spinel is also found in the cracks of coke. The stress produced by spinel crystal growth may lead to coke cracking.[23] Zhihao Zeng[24] took samples from the inside of the blast furnace during maintenance. The effect of alkali metal on coke catalysis in blast furnace was studied. Researches show that the content of alkali metal in tuyere coke is the most important factor affecting gasification reactivity, and the secondary factor is carbon chemical structure and pore structure. However, the sample amount, sampling location, and sampling depth are all restricted because it requires a sampling tube to be inserted into the blast furnace; and it is impossible to get all representative samples from the feedstock in the lower bottom part of the hearth. As for the 5,800 m^3^ super large blast furnace concerned in the present work, it is impossible to reach the central part of the deadman by using tuyere sampling. In addition, tuyere sampling is also limited to the horizontal direction at the tuyere level and unable to reach the coke below it, which prevents researchers from learning about the deterioration mechanism of the coke below tuyere level.

5800 m3 super large blast furnace of Shagang Group is one of the largest in the world. Coke samples were obtained from different positions during blast furnace intermediate repair. Cokes are taken from different heights from the edge of blast furnace to the center of deadman. The cokes in these locations are not available for tuyere sampling technology. The instruments including a polarizing microscope, X-ray diffraction (XRD) analyzer and a scan electronic microscope (SEM) were used to systematically characterize the particle size, macro-morphology, and microstructure of the coke, in an effort to explore the deterioration mechanism of coke in the hearth of the super large blast furnace.

2. Section 2.2. This manuscript determined the slag/pore ratio by calculating the ratio between their number of spots. Are the number of spots determined manually or by some software? Should this ratio be calculated by their area ratio? Please provide more details about this measurement.

Answer:

Many thanks!

How to accurately determine the percentage of reaction slag entering the coke pore has puzzled me for a long time. The slag area is very irregular and the pores are irregular. There is no way to accurately calculate the volume ratio of slag to porosity. Because I have research experience about test the optical structure of coal, I refer to Chinese standard of Microscopical Determination of vitrinite reflectance of coals (GB / T 6948-2008).

This manuscript adopts the idea of differential, dividing coke into several small cells. Each cell represents an area unit, and the slag / pore ratio can be converted by calculating the composition in the cell. This calculation method is essentially to calculate different areas.

In the process of calculation, optical microscope software is used to count automatically. Calculate the whole section of the sample. The basic principle of calculation is shown in the figure below. It can reflect the amount of slag in the coke pores.

Test steps:

（1）Point to point scanning

In the process of measurement, each measuring point is read into a small area unit, and each point represents a small area. Count after reading point data. When there are two phases in the grid, the more phases are counted.

（2）Scanning sequence

After one unit area measurement is completed, adjust the sample and carry out the scanning measurement for the next area.

（3）Proportion calculation

The porosity of unfilled slag is 0, that of slag filled is 1, and that of coke structure is 2. The meshing method is shown in Fig. 2. The slag area to pore area ratio is calculated according to the following equation.

The slag area to pore area ratio = number of slags detected/ (number of slags detected + number of pores detected) × 100%

Cell division

Scanning sequence

3. Section 3.2. Please provide the statistic analysis and error analysis of the particle size distribution measurement.

Answer:

Many thanks!

The average particle size of coke is affected by sampling method and sampling tools. The sampling site at that time is shown in the figure below. Both reviewers raised questions about the average size of coke.

As a part of the samples have been used, the remaining samples can not be retested for the average particle size of coke. Therefore, in order to avoid misleading readers with wrong conclusions, this part of the data is deleted.

4. Section 3.3. Authors were trying to analysis the slag composition of cokes extracted from BF. However, such analysis was only based on the literature review. Do you have any experimental data, such as EDS, to support your argument?

Answer:

Many thanks!

I got slag samples from blast furnace and carried out relevant tests.

This section will be support information.

The slag obtained from 5800m3 blast furnace is shown in S1. SEM and EDS analysis were carried out, as shown in Fig. 3.The results of energy spectrum analysis show that the main components of blast furnace slag are Ca, Al, Mg, Si, O, which is consistent with the results in references. A small amount of S element was also found in the slag. S element comes from coke and sulfur is absorbed by blast furnace slag.

S1 Fig. Bast furnace slag

**Ca**

**20 μm**

**P2**

**P2**

**Mg**

**Al**

**Si**

**S**

**O**

S2 Fig2. SEM and EDS analysis

5. Section 3.4. Same comment as comment 2. If this measurement was based on the ratio of number of points between slag and pore. The discussion in this section was not very convincing. An ideal measurement is the area ratio.

Answer:

Many thanks!

The answer is the same as question 2.
